# The Influence of Marital Status and Gender on Financial Well-Being

**DOI:** 10.1093/geroni/igab046.1375

**Published:** 2021-12-17

**Authors:** Jing Geng

**Affiliations:** Virginia Tech, Blacksburg, Virginia, United States

## Abstract

Research consistently documents gender differences in financial status in later life, and some also examine marital status in this regard. However, the subjective aspects of financial well-being are less well-explored, especially as this relates to both gender and marital status in the U.S. Using a gender-sensitive approach, this study examines the extent to which gender and marital status affect the financial well-being of older American adults. Different from previous studies that use only objective measures of financial well-being, this study also takes a subjective assessment in terms of financial satisfaction into account so that the role of marital status and gender in both objective measures and subjective assessments can be identified. This study uses the 2014 Health and Retirement Study and employs ordinary least squares regressions and ordinal logistic regression analyses. Examining those aged 65 and over, the sample varies from N=10,325 (financial well-being) to 4,280 (financial satisfaction). Differences in gender and marital statuses across all objective measures of financial well-being show up, with women being disadvantaged while the married (regardless of gender) being advantaged. Concerning financial satisfaction, being divorced and separated were negatively related to financial satisfaction for both men and women. These findings indicate that both marital status and gender are important indicators of financial well-being in later life.

